# Xylem and Leaf Functional Adjustments to Drought in *Pinus sylvestris* and *Quercus pyrenaica* at Their Elevational Boundary

**DOI:** 10.3389/fpls.2017.01200

**Published:** 2017-07-11

**Authors:** Laura Fernández-de-Uña, Sergio Rossi, Ismael Aranda, Patrick Fonti, Borja D. González-González, Isabel Cañellas, Guillermo Gea-Izquierdo

**Affiliations:** ^1^Department of Silviculture and Management of Forest Systems, INIA-CIFOR Madrid, Spain; ^2^Département des Sciences Fondamentales, Université du Québec à Chicoutimi Chicoutimi, Canada; ^3^Key Laboratory of Vegetation Restoration and Management of Degraded Ecosystems, Provincial Key Laboratory of Applied Botany, South China Botanical Garden, Chinese Academy of Sciences Guangzhou, China; ^4^Department of Forest Ecology and Genetics, INIA-CIFOR Madrid, Spain; ^5^Swiss Federal Institute for Forest, Snow and Landscape Research WSL Birmensdorf, Switzerland

**Keywords:** climate change, drought stress, experimental drought, phenology, sub-Mediterranean forest ecosystems, water deficit, wood anatomy, xylogenesis

## Abstract

Climatic scenarios for the Mediterranean region forecast increasing frequency and intensity of drought events. Consequently, a reduction in *Pinus sylvestris* L. distribution range is projected within the region, with this species being outcompeted at lower elevations by more drought-tolerant taxa such as *Quercus pyrenaica* Willd. The functional response of these species to the projected shifts in water availability will partially determine their performance and, thus, their competitive success under these changing climatic conditions. We studied how the cambial and leaf phenology and xylem anatomy of these two species responded to a 3-year rainfall exclusion experiment set at their elevational boundary in Central Spain. Additionally, *P. sylvestris* leaf gas exchange, water potential and carbon isotope content response to the treatment were measured. Likewise, we assessed inter-annual variability in the studied functional traits under control and rainfall exclusion conditions. Prolonged exposure to drier conditions did not affect the onset of xylogenesis in either of the studied species, whereas xylem formation ceased 1–3 weeks earlier in *P. sylvestris*. The rainfall exclusion had, however, no effect on leaf phenology on either species, which suggests that cambial phenology is more sensitive to drought than leaf phenology. *P. sylvestris* formed fewer, but larger tracheids under dry conditions and reduced the proportion of latewood in the tree ring. On the other hand, *Q. pyrenaica* did not suffer earlywood hydraulic diameter changes under rainfall exclusion, but experienced a cumulative reduction in latewood width, which could ultimately challenge its hydraulic performance. The phenological and anatomical response of the studied species to drought is consistent with a shift in resource allocation under drought stress from xylem to other sinks. Additionally, the tighter stomatal control and higher intrinsic water use efficiency observed in drought-stressed *P. sylvestris* may eventually limit carbon uptake in this species. Our results suggest that both species are potentially vulnerable to the forecasted increase in drought stress, although *P. sylvestris* might experience a higher risk of drought-induced decline at its low elevational limit.

## Introduction

The Mediterranean climate is characterized by mild, wet winters and hot, dry summers. Under climate change scenarios, an increase in the frequency and severity of drought events is projected as a result of rising temperatures without a concurrent increase in precipitation (Giorgi and Lionello, 2008; IPCC, 2013). Water has a central role in all plant physiological processes and changes in its availability could drive structural and process shifts from the leaf to the whole-tree level. A reduction in soil water availability, generally related to rainfall scarcity and often coupled with high potential evapotranspiration under warm temperatures, leads to water stress in trees, whose stem and leaf water potentials could drop beyond the boundaries of hydraulic security (McDowell et al., 2008). Plants, hence, limit canopy transpiration through stomatal closure to prevent excessive water loss and hydraulic failure (Tyree and Sperry, 1988; Bréda et al., 2006). Leaf and xylem water potentials directly influence cell turgor pressure, affecting in turn cell division and expansion (Abe et al., 2003) and, thus, radial growth and xylem architecture (Fichot et al., 2009). Likewise, potential xylem hydraulic conductivity mainly depends on the number and size of conduits, as well as on the features of the pit connections between them (Tyree et al., 1994; Hacke and Sperry, 2001; Choat et al., 2008). Wide vessels are more effective in water transport, but at the risk of suffering earlier cavitation (Sperry et al., 1994; Tyree et al., 1994). Wood anatomy is, therefore, both influenced by water availability and a key factor determining the trees’ resistance to water deficit (Sperry et al., 1994; Fichot et al., 2009). It has been generally assumed that trees under drought tend to form smaller vessels to reduce the risk of cavitation (Sperry et al., 1994). Recent work has, however, challenged this paradigm (Eilmann et al., 2009, 2011), as vulnerability to drought-induced cavitation may mostly rely on pit membrane properties rather than conduit size (Hacke and Sperry, 2001). Xylem structure and function tend, therefore, to be optimized to balance safety and water-transport efficiency (Fichot et al., 2009; Zanne et al., 2010). Moreover, there could exist a feedback between hydraulic conductance and stomatal conductance and, thus, the tree’s water use (Hacke and Sperry, 2001). Consequently, species with high vulnerability to xylem cavitation tend to exert a strong control over stomata (Brodribb et al., 2014; Klein, 2014). Regardless of the species-specific strategies to cope with drought, severe dry periods may induce hydraulic damage over time and increase sensitivity to further stress, which could eventually lead to the death of the tree (Bréda et al., 2006; McDowell et al., 2011; Anderegg et al., 2013).

Moreover, as a result of stomatal closure, photosynthetic carbon uptake and accumulation of compounds from primary metabolism are reduced (Bréda et al., 2006; McDowell et al., 2011). This implies that fewer resources can be allocated to xylem formation (Oberhuber et al., 2011). Likewise, the defensive capacity against other sources of stress can be weakened, predisposing trees to further drought stress, infections or frost damage (Bréda et al., 2006; Breshears et al., 2008; Galiano et al., 2011; McDowell et al., 2011). After a prolonged and severe drought, carbon starvation, alone or through its interplay with hydraulic failure or pathogen attacks, may ultimately cause the death of the tree (Bréda et al., 2006; McDowell and Sevanto, 2010; McDowell et al., 2011; Sala et al., 2012).

In this context, field manipulation experiments such as rainfall exclusion and irrigation have provided a better understanding of tree performance under drought and give insight on how the ongoing climate change may affect functional plant processes such as tree phenology (Ogaya and Peñuelas, 2004; Eilmann et al., 2009; Adams et al., 2015). Both the timing and duration of growth processes influence tree performance; hence, changes in phenology can significantly modulate crown and xylem development (Rossi et al., 2012; Adams et al., 2015; Pérez-de-Lis et al., 2016). Crown phenology has been widely studied through drought experiments, with delays in phenophases occurring as a result of drier conditions (Ogaya and Peñuelas, 2004; Adams et al., 2015). However, cambial phenology under experimental drought conditions has been considered only in conifer juveniles (Abe et al., 2003; de Luis et al., 2011; Balducci et al., 2013) or in boreal regions (Belien et al., 2012; D’Orangeville et al., 2013), while deciduous species and drought-prone areas have been mostly neglected (but see Klein et al., 2005 and Eilmann et al., 2011).

Tree competitive success depends on how trees adapt their functional response, xylem structure and phenology to a specific environment, defining a species potential distribution range (Tyree et al., 1994; Fonti et al., 2010; Vitasse et al., 2014). Changes in tree performance and phenology may, therefore, alter interspecific interactions and lead to shifts in species distribution, particularly at the rear edge of their distribution range. *Pinus sylvestris* L. is a Eurosiberian species, which has its southern distribution limit in the mountain ranges of the Iberian Peninsula. As a consequence of climate change, this species is expected to reduce its current distribution range, being displaced at low elevations by more drought-tolerant taxa such as sub-Mediterranean oak *Quercus pyrenaica* Willd. (Ruiz-Labourdette et al., 2012). Previous studies have already detected increased mortality of *P. sylvestris* in its lower distribution boundary (Galiano et al., 2010; Gea-Izquierdo et al., 2014). The objective of this study was to assess the responses of *P. sylvestris* and *Q. pyrenaica* under water stress conditions by analyzing their cambial and leaf phenology and wood anatomy during an experimental rainfall exclusion. Additionally, *P. sylvestris* gas exchange was measured under control and experimental conditions. The experiment, which simulated the future intensification of droughts, was located at the elevational boundary between the two species, where *P. sylvestris* is considered to be threatened by increasingly drier conditions (Gea-Izquierdo et al., 2014). We hypothesized that: (i) water deficit would induce a shortening of the xylem growing season; (ii) the effects of drought on the functional traits analyzed would be cumulative and more evident as the experiment progressed; and (iii) given its lower drought tolerance, the response of the analyzed functional traits to rainfall exclusion would be stronger in *P. sylvestris* than in *Q. pyrenaica*.

## Materials and Methods

### Study Site and Experimental Design

The study site is located in Valsaín (Segovia, Central Spain, 40° 51′ 35′′ N, 4° 3′ 52′′ W) in a north-west facing slope (15%) at an elevation of 1350 m a.s.l. It is found at the transition zone between *P. sylvestris*- and *Q. pyrenaica*-dominated woodlands and, thus, at *P. sylvestris* local dry elevational limit. The stand is composed of patches of mature *P. sylvestris* trees (sampled trees 100 ± 7 years old), younger *P. sylvestris* trees (sampled trees 43 ± 4 years old) and *Q. pyrenaica* trees (sampled trees 42 ± 1 years old). According to records obtained from the Spanish Meteorological Agency (AEMET) nearby stations, mean annual precipitation was 728 mm and mean annual temperature 9.1°C during the period 1950–2014.

To investigate the impact of drought on the physiology of the two studied species, three rainfall exclusion plots of 65–150 m^2^ were established in April 2012 based on the structure of the stand: one for *Q. pyrenaica* and two for *P. sylvestris*, one for each size class to assess the species response to the treatment regardless of tree size (**Table 1**). Rainfall was excluded from reaching the soil by fully covering it with corrugated PVC boards, which remained year-round throughout the experiment. Additionally, a 0.5 m ditch was dug and covered with plastic on the upper side of each plot to minimize downward subsurface water flow. Soil humidity was monitored in all rainfall exclusion plots and the control area with sensors set at 25 and 50 cm depth.

**Table 1:** Characteristics of the studied trees.

	*N* trees	Age (years)	DBH (cm)	Height (m)
*Q. pyrenaica* control	18	42 ± 2	16.2 ± 2.5	12.8 ± 1.5
*Q. pyrenaica* treatment	14	42 ± 1	15.4 ± 2.3	13.1 ± 1.0
*P. sylvestris* old control	7	103 ± 5	49.1 ± 7.7	23.0 ± 5.1
*P. sylvestris* old treatment	6	97 ± 7	45.7 ± 8.1	20.6 ± 1.6
*P. sylvestris* young control	9	41 ± 4	19.4 ± 4.8	14.5 ± 2.2
*P. sylvestris* young treatment	8	45 ± 2	17.5 ± 5.0	13.6 ± 1.3

*N trees, number of trees sampled over the study period; DBH, diameter at breast height.*

### Cambial Phenology and Intra-annual Growth Monitoring

Micro-cores were sampled from six trees per species and treatment, three per age class in the case of *P. sylvestris*, every 7–15 days between April and November during 2012–2014, with a total of 24 trees being monitored per year. In order to minimize inter-annual bias due to previous year punching injuries, we generally avoided using the same trees on consecutive years. Most of sampled trees subjected to rainfall exclusion were selected from the inner part of the plots to reduce the border effect. Samples were extracted with a Trephor (Rossi et al., 2006) and stored in a 70% ethanol solution at 4°C. *P. sylvestris* micro-cores sampled in 2012 were cut in sections of 10–18-μm in thickness with a sliding micro-tome. Since this technique was unsuccessful for *Q. pyrenaica*, the rest of the samples were dehydrated with increasing concentrations of ethanol and xylene, embedded in paraffin and cut with a rotary microtome in sections 8–12 μm thick. All sections were stained with Safranine O and Astra Blue, dehydrated with increasing concentrations of ethanol and xylene and mounted with either Canada balsam or a mixture of distyrene, tricresyl phosphate and xylene (DPX).

Stained sections were observed under visible and polarized light to distinguish the different xylem cell developmental stages: cambium, cell enlargement, cell-wall lignification and mature cells. For *P. sylvestris*, the number of tracheids in each phase was counted along three radial segments per date and then averaged. For *Q. pyrenaica*, photographs of the sections were taken with 10x magnification with an Olympus Colorview camera attached to a microscope. The number of cambium cells was counted and the width of earlywood and latewood increments at each cell development phase were measured along three segments with ImageJ (Schneider et al., 2012). Due to the complex anatomy of *Q. pyrenaica*’s xylem, the end of the wall-thickening phase could not be assessed on this species. Growth rates (r, in cells d^-1^ or μm d^-1^) were calculated as the ratio between the final number of tracheids or earlywood and latewood widths and the length of the cell enlargement phase, which represents a good approximation of the period of cell production.

### Leaf Phenology Monitoring

Crown phenology was recorded on the same dates of wood sampling. In 2012, leaf phenology was recorded by species at the stand level, whereas in 2013 and 2014 it was recorded individually on the trees used to study cambial phenology. The dates of bud-burst, end of leaf and needle elongation and, in *Q. pyrenaica*, the beginning of autumn leaf discolouration, were identified.

### Wood Anatomy

To avoid bias in inter-annual comparisons due to the selection of different trees each study year, a micro-core including the tree rings belonging to the period 2011-2014 was taken from every monitored tree (**Table 1**) in November 2014 to assess the cumulative effect of the rainfall exclusion treatment on wood anatomy. Anatomical sections were prepared according to the abovementioned protocol and photographed with 10x magnification and a resolution of 2.2 pixels/μm. Cell-wall thickness, lumen area and earlywood and latewood widths were measured along three radial rows in *P. sylvestris*. For *Q. pyrenaica*, earlywood and latewood widths and the area of earlywood vessels with a lumen area > 7500 μm^2^ were measured. Earlywood vessel area was additionally measured in all the section photographs taken for the analysis of cambial phenology. All measurements were carried out with ImageJ (Schneider et al., 2012).

Given the different functionality of earlywood (EW) and latewood (LW), the ratio between latewood width and ring width (LW/RW) was calculated for both species to assess whether rainfall exclusion influenced the proportion of each type of wood. Lumen areas were used to estimate vessel and tracheid radii, which were in turn used to calculate the hydraulic diameter (D_H_). D_H_, computed as Σd^5^/Σd^4^, where d is conduit diameter, is proportional to the hydraulic conductance given by the Hagen–Pouseuille law and, thus, a proxy of xylem hydraulic conductivity (Sperry et al., 1994).

### *Pinus sylvestris* Physiological Measurements

Morning net photosynthetic rate (*A*; μmol m^-2^ s^-1^) and stomatal conductance to water vapor (*g*_s_; mol m^-2^ s^-1^) and midday leaf water potential (Ψ; MPa) were recorded for *P. sylvestris* in late July or early August between 2012 and 2014. Six trees per treatment (three per age class) were measured. The inaccessibility of leaves of *Q. pyrenaica* trees at the rainfall exclusion plot precluded the possibility to carry out measurements in this species. Gas exchange measurements were performed on current-year needles exposed to direct sunlight with an LCpro+ portable photosynthesis system (ADC BioScientific Ltd., Hoddesdon, Herts, United Kingdom). Leaf water potential was measured with a Scholander pressure chamber (PMS Instrument Co. 7000, Corvallis, OR, United States). For each tree, a subsample of the measured needles was scanned to measure needle area. Subsequently, needles were dried and weighed to obtain their specific leaf area (SLA; cm^2^ g^-1^), calculated as the ratio between needle area and dry mass. SLA was used to recalculate net photosynthetic rate and stomatal conductance on a leaf area basis.

Additionally, collected needles were ground to analyze carbon and nitrogen elemental concentration and stable carbon isotope ratio (δ^13^C). Samples of 3–4 mg were analyzed using a PDZ Europa ANCA-GSL elemental analyzer interfaced to a PDZ Europa 20-20 isotope ratio mass spectrometer (Sercon Ltd., Cheshire, United Kingdom). The average δ^13^C analytical precision was 0.1‰. δ^13^C data were transformed to discrimination (Δ) following Farquhar et al. (1989):

(1) $$\text{Δ}\approx \frac{{\text{δ}}^{13}{\text{C}}_{\text{a}}-{\text{δ}}^{13}\text{C}}{1+{\text{δ}}^{13}\text{C}}$$

Source air δ^13^C (δ^13^C_a_) data were obtained from NOAA Earth System Research Laboratory (White et al., 2015).

Needle carbon and nitrogen concentrations on a mass basis were used to calculate C/N ratio and photosynthetic nitrogen use efficiency (PNUE; μmol g^-1^ s^-1^), which is the ratio between net photosynthetic rate and leaf nitrogen content.

### Data Analysis and Statistics

For each sampling date, phenological observations were converted into presence/absence (binary) data. Logistic functions were used to estimate the day of the year (DOY) at which each cambial and foliar developmental phase began and ended. Preliminary analysis showed no differential effect of the treatment between *P. sylvestris* tree sizes. Hence, data were pooled by species to increase sample size and, thus, statistical robustness. Differences in phenology between treatments were assessed fitting generalized linear mixed models (GLMM) with a binomial distribution and a random intercept associated with tree. The assessed fixed factors were DOY, year (to account for inter-annual climatic variability), treatment and the interaction ‘treatment × year.’ The Akaike Information Criterion (AIC) was used to compare the different GLMMs fitted. Lower values of AIC indicate a better fit. A model was considered to have a significantly higher explanatory power than another when the difference in AIC (ΔAIC) between models was ≥ 2 (Burnham and Anderson, 2002). If ΔAIC < 2, the difference among models was considered non-significant and, therefore, the more parsimonious model (i.e., with fewer variables) was selected.

Because data were not normally distributed and measures were repeated within individuals, differences between treatments in the number of cambial cells, total growth, growth rates and wood anatomical features were assessed using GLMMs with a Gamma distribution and an identity link function, except for *Q. pyrenaica* growth rates, for which a log link function was used. We fitted GLMMs with the fixed effects treatment, year and the interaction ‘treatment × year’ and a random intercept to account for correlation within trees. If the best model, given by its AIC, included the interaction ‘treatment × year,’ we tested the differences in specific years using Tukey contrasts. Differences in gas exchange and leaf chemical properties between treatments were assessed using Mann–Whitney–Wilcoxon test.

All analyses were carried out with R version 3.1.1 (R Core Team, 2014). GLMMs were fit with the packages “lme4” (Bates et al., 2014) and “glmmADMB” (Fournier et al., 2012). Multiple mean comparisons (Tukey contrasts) were performed with the package “multcomp” (Hothorn et al., 2008).

## Results

**FIGURE 1:**
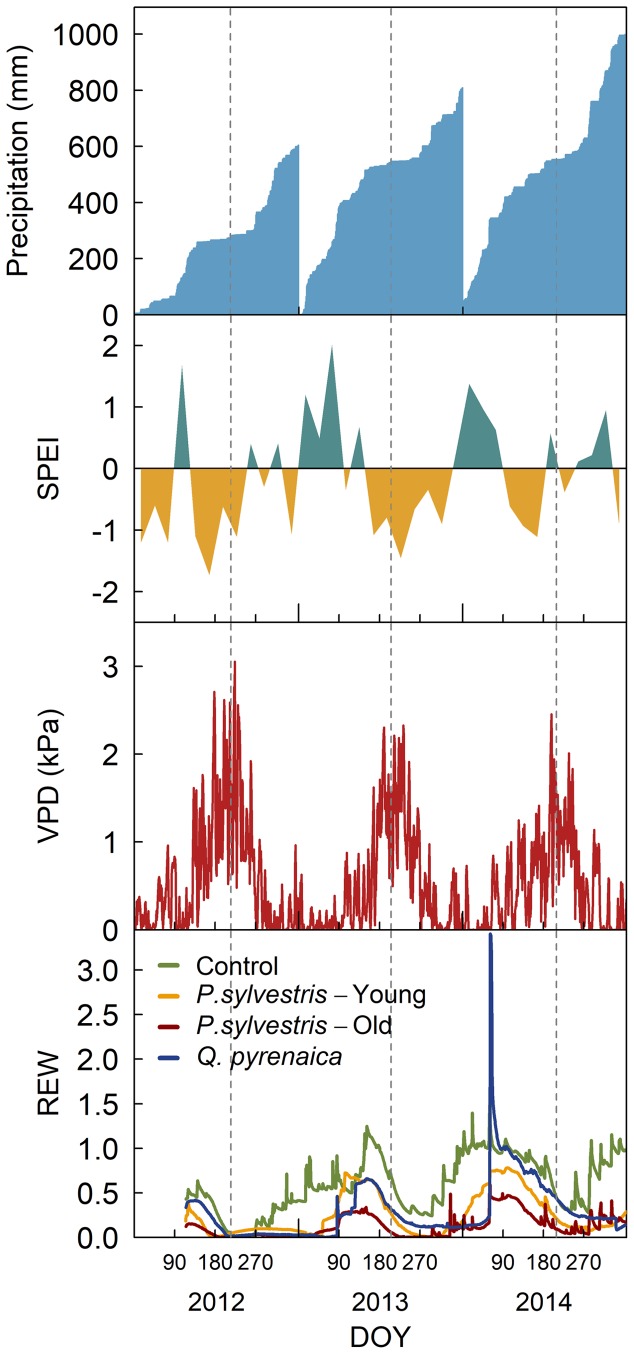
Cumulative precipitation, monthly standardized precipitation-evapotranspiration index (SPEI), daily vapor pressure deficit (VPD) and daily relative extractable water (REW) during the study period. REW is given for each of the rainfall exclusion plots and the control area. Vertical dashed lines mark the days in which gas exchange measurements were taken.

Annual mean temperatures and total precipitation during the study years were, respectively, 9.8°C and 604 mm in 2012, 9.1°C and 808 mm in 2013 and 10.1°C and 998 mm in 2014, making 2012 a drier than average year, 2013 an average year and 2014 a warmer but wetter year than the average (**Figure 1**). Summer was characterized by dry conditions, particularly in 2012 and 2013, as indicated by the negative monthly standardized precipitation-evapotranspiration index (SPEI; Vicente-Serrano et al., 2010), high vapor pressure deficit (VPD) – that reached punctual values of 3 kPa in 2012 – and low soil relative extractable water (REW; Granier, 1987) during that period of the year (**Figure 1**). Compared to control, the rainfall exclusion reduced soil humidity in the top 50 cm by 30% in the young-tree *P. sylvestris* rainfall exclusion plot, 40% in the *Q. pyrenaica* plot and 80% in the old-tree *P. sylvestris* plot (**Figure 1**).

### Cambial and Leaf Phenology

**FIGURE 2:**
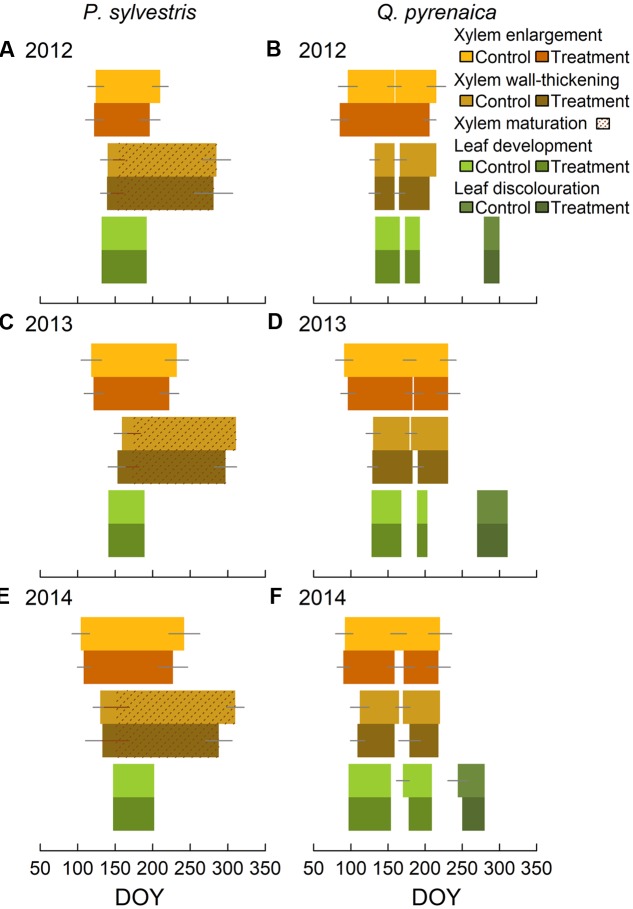
*Pinus sylvestris* **(A,C,E)** and *Quercus pyrenaica* **(B,D,F)** phenological phases in control and trees subjected to rainfall exclusion. Gray lines mark standard deviations. *Q. pyrenaica* cambial phenology is separated by earlywood and latewood.

In *Pinus sylvestris*, the number of cambial cells during dormancy was 6 ± 2, whereas active cambium had 10 ± 3 cells (Supplementary Figure S1). The number of cambium cells, both active and in dormancy, was significantly lower in trees subjected to rainfall exclusion only in 2013. Xylem formation started between mid-April and early-May (DOYs 105-122), with cell wall-thickening starting on average 2–5 weeks later (**Figure 2** and Supplementary Figure S1). The first cells completed maturation between the end of May and the end of June (DOYs 142-176), depending on the year (**Figure 2**). Xylem enlargement ended between mid-July and early-September (DOYs 203–234), depending on the year. Ring maturation was completed in October to early-November (DOYs 283–299). No significant differences between treatments were found in the beginning of the enlargement or wall-thickening phases (**Table 2**), whereas rainfall exclusion significantly influenced the end of both phases, with trees subjected to rainfall exclusion finishing growth one to 3 weeks earlier than control trees (**Table 2** and **Figure 2**). The strength of the effect depended, however, on the year (**Table 2**).

**Table 2:** Effect of treatment (T) and year (Y) on the onset and cessation of *Pinus sylvestris* phenological phases as given by the differences in the Akaike Information Criterion (ΔAIC) of the fitted GLMMs.

	Phase onset				Phase cessation		
Model	E	W	M	ND	E	W	ND
DOY	13.93	18.50	34.76	38.60	67.83	16.02	53.04
DOY + Y	**0.00**	**0.00**	**0.00**	**0.00**	4.61	5.88	**0.00**
DOY + T	14.96	20.30	36.73	42.11	64.81	13.47	31.11
DOY + T + Y	4.64	1.99	2.28	23.34	**0.00**	3.15	19.21
DOY + T + Y + (T × Y)	7.01	1.74	3.39	42.50	2.65	**0.00**	35.91

*ΔAIC was calculated as the difference between the AIC of the model being assessed and the AIC of the model with the lowest AIC (i.e., the model with ΔAIC = 0). The best model is marked in bold. E, enlargement phase; W, wall-thickening phase; M, mature cells; ND, needle development; DOY, day of the year.*

Moreover, one of the old pine trees subjected to drought died between the 2013 and 2014 growing seasons. During 2013, cambium was mostly inactive and formed at most four tracheids, which in many cases did not lignify (Supplementary Figure S2). Cambial inactivity occurred before crown damage was evident. Since the growth of this tree before the rainfall exclusion was applied did not differ from that of other trees in the stand, its death was likely a direct consequence of the intensification of drought by the rainfall exclusion treatment. Furthermore, the presence of mistletoe (*Viscum album* L.) on this tree might have exacerbated the rainfall exclusion effect.

In *Q. pyrenaica*, the number of cambium cells during dormancy was 8 ± 1, whereas active cambium had 10 ± 2 cells, with no significant differences between treatments (Supplementary Figure S3). Xylem growth presented a bimodal pattern, corresponding to the peaks in earlywood vessel and latewood formation (Supplementary Figure S3). Earlywood growth onset occurred between late-March and early-April (DOYs 91–94) and vessel lignification started 1–8 weeks later, depending on the year (**Figure 2**). Latewood development started between June and early-July (DOYs 160–182) and its lignification began up to 2 weeks later (**Figure 2**). Latewood enlargement ceased at the end of July-mid-August (DOYs 211–231). No significant differences were observed between treatments in *Q. pyrenaica* cambial phenology (**Table 3** and **Figure 2**).

**Table 3:** Effect of treatment (T) and year (Y) on the onset and cessation of *Quercus pyrenaica* phenological phases.

	Phase onset							Phase cessation			
Model	EW E	EW W	LW E	LW W	LD1	LD2	ALD	EW E	LW E	LD1	LD2
DOY	**0.00**	20.45	**0.20**	**0.00**	49.76	**0.00**	38.16	**0.00**	**0.86**	16.25	26.74
DOY + Y	1.46	**0.00**	0.00	38.50	**0.00**	1.82	**0.00**	2.20	0.00	**0.00**	**0.00**
DOY + T	1.72	22.52	0.71	19.18	51.68	9.13	53.68	18.02	2.86	17.62	36.80
DOY + T + Y	3.39	1.22	1.42	10.92	2.00	6.69	2.00	5.56	2.86	1.09	2.00
DOY + T + Y + (T × Y)	4.95	3.33	7.64	33.95	4.00	20.39	3.67	14.71	4.89	3.06	4.00

*Differences in the Akaike Information Criterion (ΔAIC) of the fitted GLMMs were calculated as the difference between the AIC of the model being assessed and the AIC of the model with the lowest AIC (i.e., the model with ΔAIC = 0). The best model (i.e., the one with fewer number of parameters of those with ΔAIC < 2) is marked in bold. EW E, enlarging earlywood; EW W, wall-thickening and mature earlywood; LW E, enlarging latewood; LW W, wall-thickening and mature latewood; LD1, spring leaf development; LD2, Summer leaf development; ALD, autumn leaf discoloration; DOY, day of the year.*

Budburst occurred synchronously among individuals of each species within each study year; in *P. sylvestris*, between DOYs 132 and 147, depending on the year (**Figure 2**). In *Q. pyrenaica*, budburst occurred between DOYs 97 and 133, depending on the year, with a second leaf sprouting occurring between DOYs 173 and 189 (**Figure 2**). Oak leaf discoloration started between DOYs 233 and 279, depending on the year. Leaf phenology was unaffected by rainfall exclusion in both species (**Tables 2**, **3**).

### Growth Rates, Total Growth and Wood Anatomy

**FIGURE 3:**
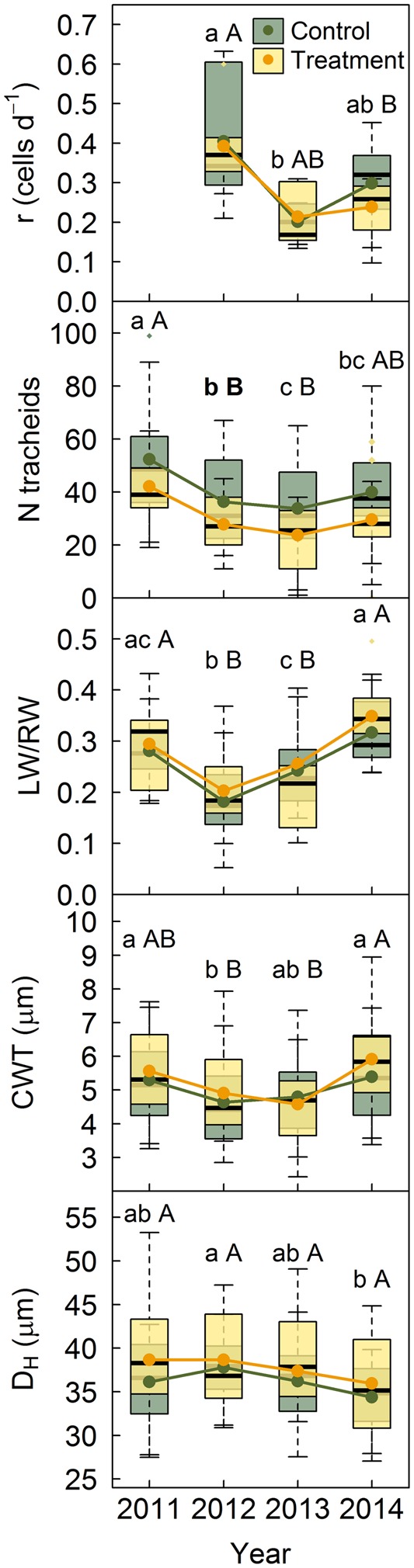
Growth rates and anatomical properties for *P. sylvestris* in control trees and trees subjected to rainfall exclusion. Anatomical traits were also analyzed for the year prior to the establishment of the experiment. Lower case letters indicate differences among years in control trees and capital letters indicate differences among years in treated trees. Bold letters indicate differences between treatments within each year. r, growth rate; N tracheids, number of tracheids; LW/RW, proportion of latewood as a function of ring width; CWT, cell-wall thickness; D_H_, hydraulic diameter.

No significant differences were found between treatments in *P. sylvestris* growth rates for any of the studied years (**Figure 3**). For both treatments, growth rates were highest in 2012, the driest year. Control trees had significantly more tracheids per ring than trees subjected to rainfall exclusion only in 2012 (**Figure 3**). None of the other anatomical features assessed (LW/RW ratio, D_H_ and cell-wall-thickness) showed differences between treatments (**Figure 3**). However, the ratio LW/RW was lowest in 2012, the driest year, and highest in 2014, the wettest. Cell-wall thickness behaved similarly, whereas D_H_ decreased in 2014, although this drop was only significant for control trees.

**FIGURE 4:**
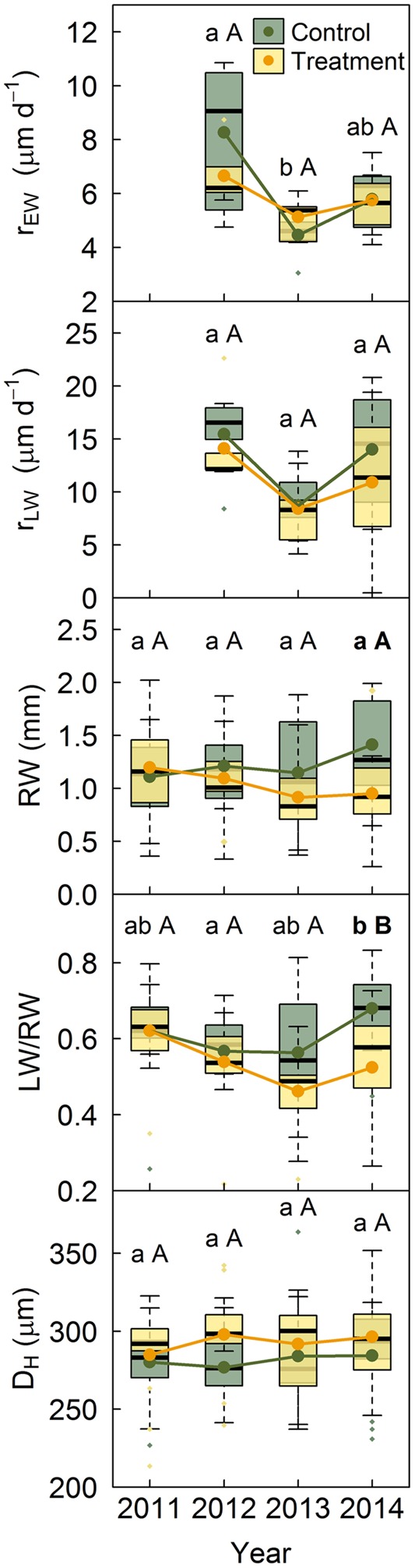
*Quercus pyrenaica* growth rates and anatomical properties per treatment. Anatomical traits include the year prior to the establishment of the experiment. Lower case letters indicate differences among years in control trees and capital letters indicate differences among years in treated trees. Bold letters indicate differences between treatments within each year. r_EW_, earlywood growth rate; r_LW_, latewood growth rate; LW/RW, proportion of latewood as a function of ring width; D_H_, hydraulic diameter.

No significant differences were found between treatments in *Q. pyrenaica* growth rates, neither for EW nor for LW, although LW rates tended to be lower under rainfall exclusion (**Figure 4**). Both growth rates were lowest in 2013, although this reduction was only significant for EW growth rates between 2012 and 2013 in control trees. Ring width and the LW/RW ratio decreased in trees subjected to rainfall exclusion, with differences between treatments becoming significant in 2014 (**Figure 4**). D_H_ did not differ between treatments or years in this species.

### *Pinus sylvestris* Gas Exchange

**FIGURE 5:**
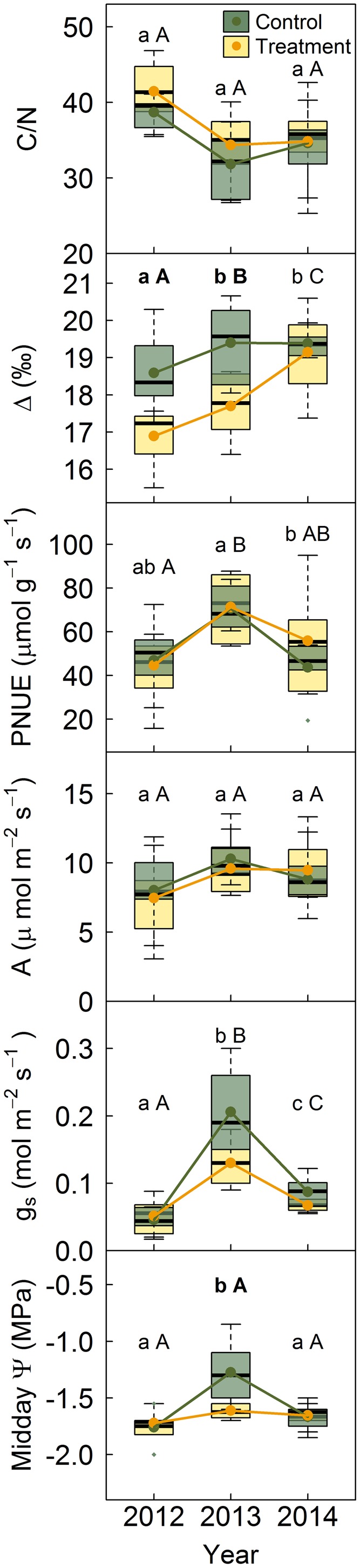
*Pinus sylvestris* needle chemical properties, morning gas exchange and midday leaf water potentials per treatment. Lower case letters indicate differences among years in control trees and capital letters indicate differences among years in treated trees. Bold letters indicate differences between treatments. C/N, C/N concentration ratio; PNUE, photosynthetic nitrogen use efficiency; Δ, carbon discrimination; A, morning photosynthetic rate; g_s_, morning stomatal conductance; Midday Ψ, midday leaf water potential.

No significant differences were found between treatments or years in needle C/N ratio (**Figure 5**). However, needle Δ significantly increased along the study period in both treatments and was significantly lower in trees under rainfall exclusion than in control trees in 2012 and 2013. No differences in PNUE, *A* or *g*_s_ were observed between treatments, although *g*_s_ was marginally significantly lower (*P* = 0.067) in trees subjected to rainfall exclusion than in control trees in 2013 (**Figure 5**). PNUE was highest in 2013. *A* did not suffer significant inter-annual variation, although it was marginally significantly lower (*P* = 0.065) in 2012, the driest year of the three studied, particularly under rainfall exclusion. *g*_s_ greatly varied among years, especially in control trees, being lowest in 2012 and highest in 2013. Midday Ψ was significantly more negative in treated than in control trees in 2013. Leaf water potentials barely differed among measurement dates under the rainfall exclusion treatment but were significantly less negative in control trees in 2013 (**Figure 5**).

## Discussion

### Drought Affects Cambial Rather Than Leaf Phenology

Xylem formation was significantly shortened under the rainfall exclusion treatment in *P. sylvestris* (**Figure 2**). Conversely, the onset of xylogenesis was not affected by rainfall exclusion in either of the two species studied, indicating that other factors could be involved in the resumption of cambial activity, such as temperatures or photoperiod (Rossi et al., 2008; Pérez-de-Lis et al., 2016). Thus, as hypothesized, the length of the growing season was shorter under drought, particularly in *P. sylvestris*, which confirmed its higher sensitivity to drought than the studied sub-Mediterranean oak. Concurring with our results, Klein et al. (2005) and Eilmann et al. (2011) found that trees had shorter growing seasons under drought due to an earlier cessation of growth rather than a later onset in *Pinus halepensis* and *P. sylvestris*, respectively. This earlier cessation of xylogenesis is consistent with a shift in the allocation of resources under stress, favoring carbon storage, bud formation or fine root growth over stem secondary growth (Waring, 1987; Eilmann et al., 2011; Wiley et al., 2013). On the other hand, Belien et al. (2012) and D’Orangeville et al. (2013) did not observe an effect of rainfall exclusion on cambial phenology in *Picea mariana* and *Abies balsamea* located in boreal forests.

Conversely, no differences were found in leaf phenology as a result of rainfall exclusion for either of the study species (**Figure 2**). Likewise, Adams et al. (2015) found that *Juniperus monosperma* was unaffected by a drought experiment, although they observed a delay in *Pinus edulis* leaf phenology under the treatment during an already dry year. Ogaya and Peñuelas (2004) found a delay in *Arbutus unedo* crown phenophases as a result of a drought experiment, but not in *Quercus ilex* and *Phillyrea latifolia*. Concurring with our study, Klein et al. (2005) found no changes in *P. halepensis* needle phenology due to irrigation, while the treatment strongly affected stem growth period length. Therefore, the severity of drought necessary to trigger shifts in leaf phenology seems to be species-specific (Peñuelas et al., 2004), but stronger than that modifying cambial phenology.

### Species-Specific Plasticity in Anatomical Traits in Response to Drought

Unlike our hypothesis, no cumulative effect was observed in *P. sylvestris* anatomical traits during the 3-year rainfall exclusion; they were, however, influenced by year-to-year climatic variability (**Figure 3**). Control trees had a significantly higher number of tracheids than treated ones only in 2012, the driest year. Given the lack of differences in growth rates between treatments, this is probably due to tree-intrinsic characteristics, such as the number of cambial cells, and treatment-derived effects, such as the shorter growing season found in trees subjected to drought. Moreover, the number of tracheids was significantly lower in 2012 and 2013, the driest years. Experiments performed in different conifer species have found an increase in cell production in irrigated compared to non-irrigated trees (Abe et al., 2003; Eilmann et al., 2009; de Luis et al., 2011; Balducci et al., 2013). On the other hand, Belien et al. (2012) did not find differences in the number of tracheids formed under drought and D’Orangeville et al. (2013) only found differences in tree ring size on the second year of the experiment. This variability may suggest that the response of cambial production to drought is highly dependent on its severity and duration. Drought episodes have been found to strongly influence secondary growth even after several years, either by cumulative hydraulic damage (Anderegg et al., 2013) or by affecting the recovery of the canopy and its indirect effect on carbon reserves due to reduced photosynthetic carbon uptake (Galiano et al., 2011). Thus, a stronger effect of drought on tracheid production may be expected after prolonged exposure to rainfall exclusion.

Similarly to Balducci et al. (2013), we did not observe differences in *P. sylvestris* wood anatomy between treatments (**Figure 3**). Nonetheless, hydraulic diameter tended to be higher and cell-wall thickness lower in the driest year, particularly in control trees, which resulted in a greater proportion of earlywood, indicating that anatomical traits respond to climatic variability. By contrast, Belien et al. (2012) and D’Orangeville et al. (2013) found that the tracheids of trees under drought had smaller lumen size and thicker cell walls than those of control trees. Eilmann et al. (2009, 2011), however, also found greater lumen diameters under drought in *P. sylvestris*. Therefore, this species may tend to produce fewer tracheids under dry conditions without reducing water transport efficiency due to the greater proportion of large-lumen cells. This enhancement in hydraulic diameter may not, however, necessarily increase its vulnerability to cavitation as drought-induced cavitation mostly depends on pit membrane properties, rather than conduit diameter (Hacke and Sperry, 2001; Martínez-Vilalta et al., 2009).

On the other hand, the more-drought tolerant *Q. pyrenaica* did not show differences in earlywood vessel hydraulic diameter between treatments or years (**Figure 4**), whilst other studies in sub-Mediterranean oaks have found both wider (Corcuera et al., 2006) and narrower (Eilmann et al., 2009) earlywood vessels under drought. However, consistent with these studies, we found that *Q. pyrenaica* trees subjected to rainfall exclusion suffered an increasing reduction in latewood width along the study period (**Figure 4**). This hypothesized cumulative effect of the rainfall exclusion treatment might have been more evident in this species than in *P. sylvestris* due to the heavy reliance of ring-porous species on stored carbohydrates to form their annual rings (Michelot et al., 2012). This could have intensified the rainfall exclusion influence as reserves may have not been fully replenished throughout the experiment. Although rainfall exclusion did not affect the size of earlywood vessels and, thus, the capacity of the main conductive system remained unaltered, the reduction in latewood under drought could ultimately challenge hydraulic performance in this species as latewood vessels provide water transport for several years once earlywood vessels become non-functional due to cavitation (Granier et al., 1994).

The reduction in the number of tracheids formed by *P. sylvestris* in drier years suggests that the allocation of resources to xylem was reduced under drought. The observed increase in hydraulic diameter under drought could have, however, partially offset this reduction in carbon allocation to xylem formation by likely enhancing tracheid water transport efficiency. By contrast, *Q. pyrenaica* maintained earlywood vessel size but reduced latewood width under rainfall exclusion. Therefore, this species invested less carbon in xylem formation but at a greater risk of hydraulic failure, particularly in extremely dry years when latewood functionality could be critical to maintain hydraulic sufficiency (Granier et al., 1994; Corcuera et al., 2006). Thus, although with different effects on their hydraulic system, our observations suggest that both species followed carbon-saving strategies in xylem formation under drought. This is consistent with studies that indicate that trees adjust to drought by first limiting secondary growth rather than carbon assimilation (Waring, 1987; McDowell et al., 2008; Oberhuber et al., 2011; Fatichi et al., 2014; Adams et al., 2015). This limitation was more evident in the old *P. sylvestris* tree that died during the experiment, which barely had any cambial activity prior to its death while the crown was still functional. Albeit an isolated event within our plots, water stress-related mortality is not unprecedented within the study area (Gea-Izquierdo et al., 2014). The death of this tree under the rainfall exclusion treatment could, therefore, portend high vulnerability to drought-induced mortality in *P. sylvestris* at its dry limit (Martínez-Vilalta and Piñol, 2002; Vilà-Cabrera et al., 2011).

### Tight Stomatal Control in *Pinus sylvestris* Determines Its Response to Drought

PNUE was unaltered by the rainfall exclusion treatment; it suffered, however, high year-to-year variability, suggesting a climatic influence on this variable (**Figure 5**). PNUE inter-annual changes followed those in *g_s_* and, to a lesser extent, *A*. These variables were highest in 2013, despite 2014 was the wettest year, which may be due to REW was higher on the dates measurements were taken in 2013 than in the other two study years (**Figure 1**). The pattern followed by these variables is consistent with studies that show that decreases in *g_s_* reduce PNUE, while increase intrinsic water use efficiency (iWUE; Limousin et al., 2015).

Needles of trees under the rainfall exclusion treatment had lower Δ and, thus, higher iWUE, than control trees (**Figure 5**). This effect of rainfall exclusion on the more integrative δ^13^C-derived iWUE was, however, less evident in instantaneous gas exchange measurements, which only showed marginally significant differences between treatments in *A* and *g_s_*. Due to the non-linearity of the relationship between *A* and *g_s_*, stomatal closure causes a proportionally greater decrease in transpiration than in photosynthesis (Limousin et al., 2015). This may explain the higher response to the rainfall exclusion treatment and inter-annual variability observed in *g_s_* and Δ, both lowest in the driest year, than in *A*. Nonetheless, photosynthetic rates are tightly coupled to stomatal control of water use and, therefore, long-term stomatal limitations could significantly alter the tree’s carbon reserves (Galiano et al., 2011).

Midday leaf water potentials followed changes in *g_s_* in control trees whereas they remained more negative in trees subjected to rainfall exclusion, being the difference between treatments significant in 2013 (**Figure 5**). Nevertheless, midday Ψ observed at our site were above those reported to cause significant xylem cavitation (Martínez-Vilalta and Piñol, 2002; Poyatos et al., 2008) and the absolute minimum values reported for the species (-2.5 MPa; Martínez-Vilalta et al., 2009). Hence, thanks to the tight control exerted by stomata to avoid excessive water loss and, consequently, prevent severe damage to the hydraulic system (Irvine et al., 1998; Poyatos et al., 2008), *P. sylvestris* may afford increasing current year tracheid size under dry conditions, as observed in this and other studies (Eilmann et al., 2009, 2011), without increasing the risk of drought-induced cavitation.

### Challenges of Rainfall Exclusion Experiments

Rainfall exclusion and irrigation experiments have been central for improving our understanding of how tree physiology responds to drought (Beier et al., 2012). Our rainfall exclusion treatment was successful in decreasing soil water content (**Figure 1**). However, most of the studied traits showed non-significant differences between treatments, despite they responded to inter-annual rainfall variability. This lack of significant differences might be due to trees being able to cope with imposed drought by absorbing water from deeper soil layers (Belien et al., 2012), from roots reaching beyond the exclusion area or from intercepted rainfall (Breshears et al., 2008), minimizing the effect of the treatment. Additionally, the field and laboratory intensive requirements characteristic of cambial phenology, wood anatomy and gas exchange studies resulted in small sample sizes, which probably obscured statistically significant effects of the rainfall exclusion treatment (Metz et al., 2016). Despite these shortcomings, our study provides valuable information on how these traits respond to an intensification of natural droughts. Although the focus of this study was the response of cambial phenology and wood anatomy to drought in the two study species, with the additional insight provided by leaf phenology in both species and gas exchange measurements in *P. sylvestris*, complementing these measurements with other variables, such as leaf chlorophyll, partial dehydration or leaf area index, would provide a more integral understanding of tree response to drought. Nonetheless, the results reported showed that secondary growth responded more strongly to the treatment than the canopy variables assessed, which would be consistent with the shift in resource allocation under drought stress observed in other studies (Waring, 1987; Oberhuber et al., 2011; Fatichi et al., 2014).

## Conclusion

The drought experiment affected xylem formation in both *P. sylvestris* and *Q. pyrenaica*. *P. sylvestris* responded to rainfall exclusion by completing xylem formation earlier than control trees. Leaf phenology was, however, unaffected by rainfall exclusion, evidencing that cambial phenology is more sensitive to drought than leaf phenology and, thus, a higher drought intensity is required to drive changes in leaf phenology than in xylogenesis processes. Furthermore, *P. sylvestris* formed fewer, although larger tracheids under drought, whereas *Q. pyrenaica* reduced its latewood throughout the rainfall exclusion experiment. Altogether, these findings are consistent with a shift in the allocation of resources under drought stress, favoring other sinks, such as carbon storage or bud formation, over xylem growth (Waring, 1987; Oberhuber et al., 2011; Wiley et al., 2013; Fatichi et al., 2014; Adams et al., 2015). Moreover, *P. sylvestris* stomatal conductance presented lower values under rainfall exclusion in order to avoid excessive water loss, maximize intrinsic water use efficiency and, ultimately, maintain water potentials above cavitation-risk levels. This suggests that carbon uptake may also be compromised in this species by increasing drought stress, eventually affecting long-term carbohydrate storage. Therefore, the outcomes from our experiment indicate that both species are potentially vulnerable to the increasing drought stress expected with the ongoing climate change. However, mortality events previously observed in the study area (Gea-Izquierdo et al., 2014), together with the stronger phenological response and the death of an old pine under rainfall exclusion, may suggest a higher risk of drought-induced decline in *P. sylvestris* at its lower elevational limit.

## Author Contributions

GGI, IA, IC, LFU, PF, and SR conceived the ideas and designed methodology; BGG, GGI, IA, and LFU collected the data; LFU and SR analyzed the data; LFU led the writing of the manuscript. All authors contributed critically to the drafts and gave final approval for publication.

## Conflict of Interest Statement

The authors declare that the research was conducted in the absence of any commercial or financial relationships that could be construed as a potential conflict of interest.
