# Supplementary material for: Xylem and Leaf Functional Adjustments to Drought in Pinus sylvestris and Quercus pyrenaica at Their Elevational Boundary
Source: Front Plant Sci. 2017 Jul 11;8:1200. doi: 10.3389/fpls.2017.01200 (PMC5504171; doi:10.3389/fpls.2017.01200)
Supplement: Supplementary file 1 [file Image_1.pdf]

Supplementary Material

Xylem and leaf functional adjustments to drought in *Pinus sylvestris* and *Quercus pyrenaica* at their elevational boundary

Laura Fernández-de-Uña*, Sergio Rossi, Ismael Aranda, Patrick Fonti, Borja D. González-González, Isabel Cañellas, Guillermo Gea-Izquierdo

*** Correspondence:** Laura Fernández de Uña: laura.fernandezdeuna@gmail.com

1 Supplementary Figures

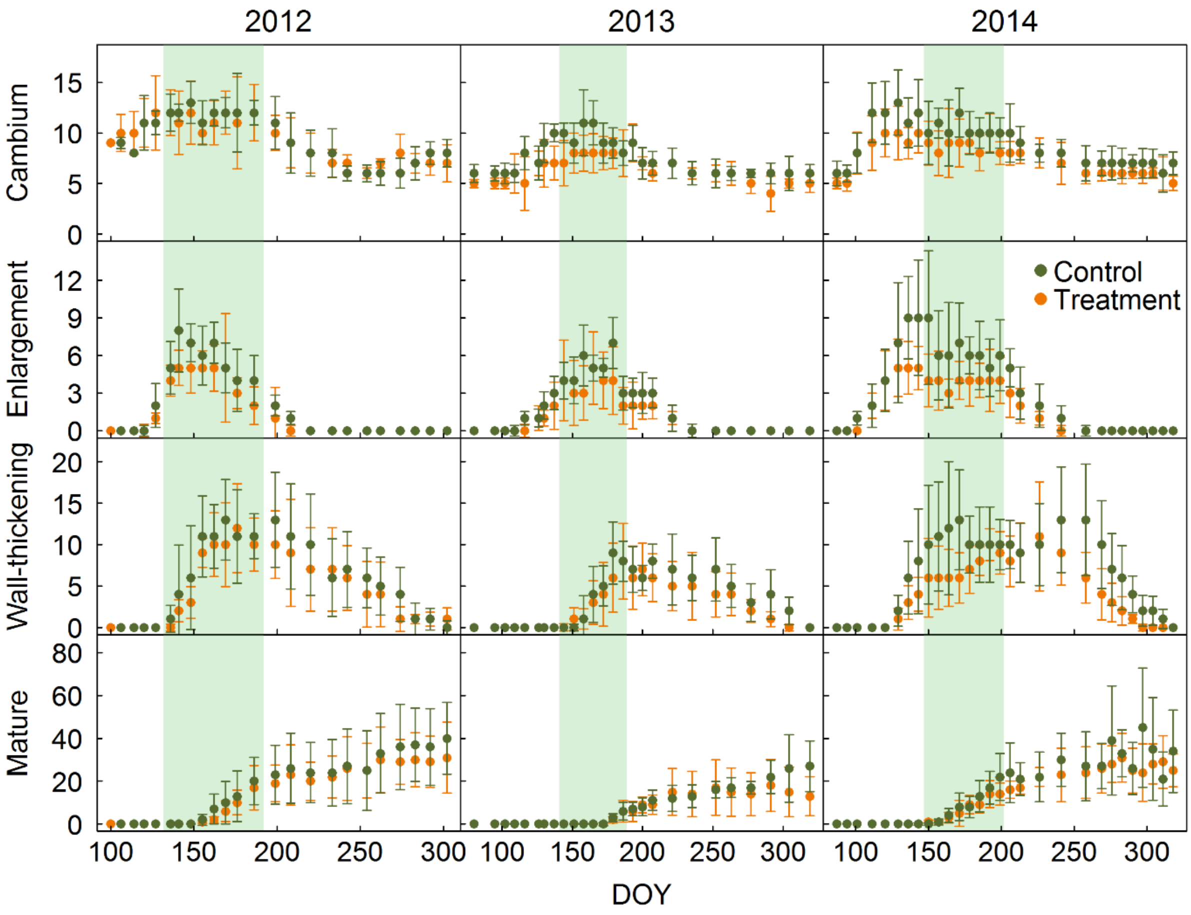

Supplementary Figure 1. *Pinus sylvestris* intra-annual growth dynamics (number of tracheids at each developmental stage) in control and trees subjected to rainfall exclusion. Vertical bars represent standard deviations. Shaded areas mark the period of needle development.

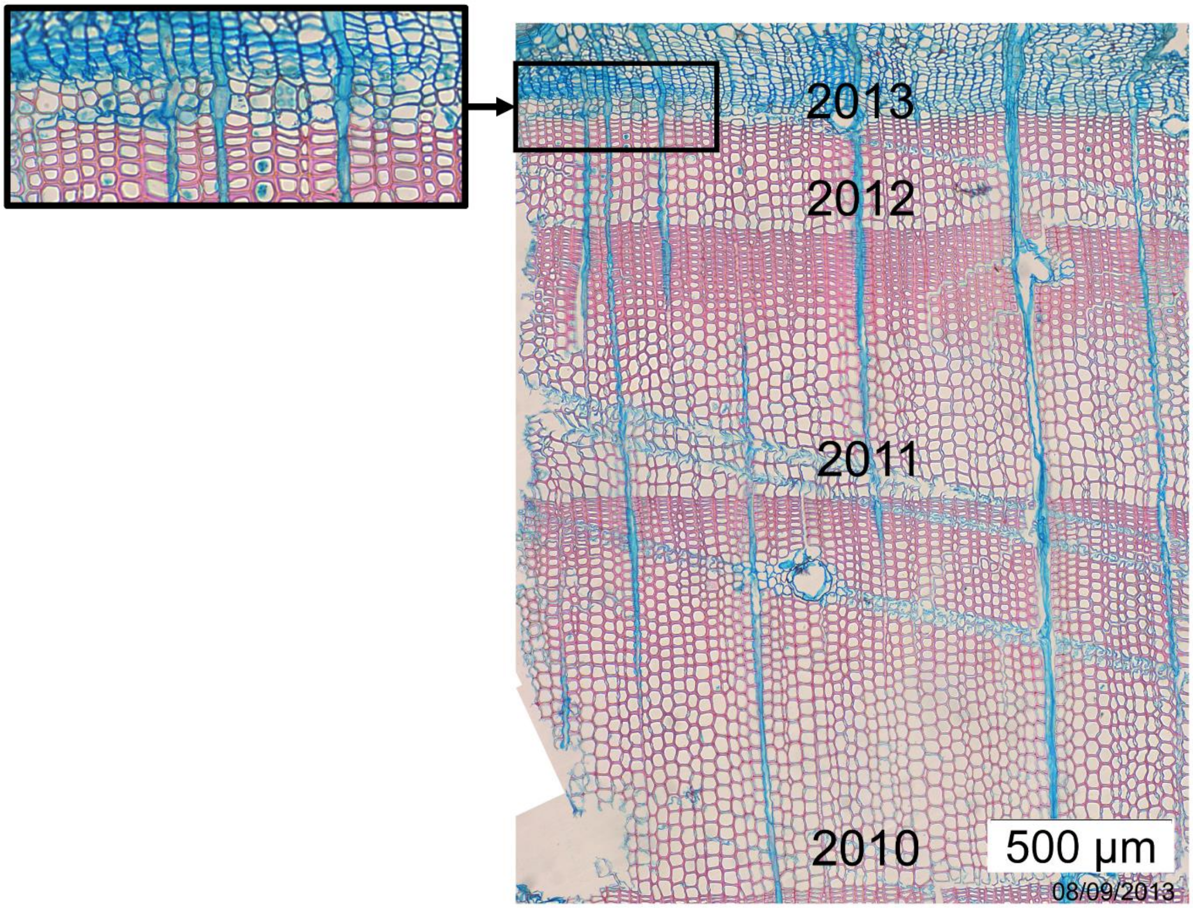

Supplementary Figure 2. Section of the dying *Pinus sylvestris* tree showing inactive cambium and non-lignified cells formed during the 2013 growing season.

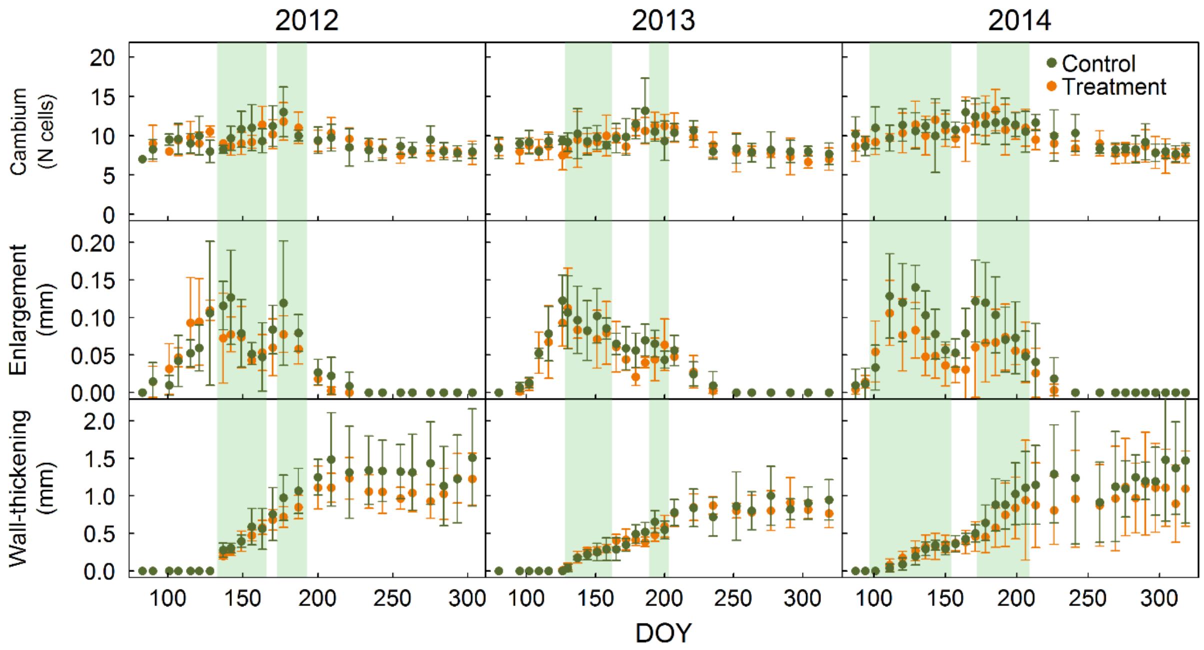

Supplementary Figure 3. *Quercus pyrenaica* intra-annual growth dynamics (length of enlarging and wall-thickening/mature xylem) for control and trees subjected to rainfall exclusion. Vertical bars represent standard deviations. Shaded areas mark the periods of leaf development.
